# Fabrication of Ordered SnO_2_ Nanostructures with Enhanced Humidity Sensing Performance

**DOI:** 10.3390/s17102392

**Published:** 2017-10-20

**Authors:** Wei Li, Juyan Liu, Chao Ding, Gang Bai, Jie Xu, Qingying Ren, Jinze Li

**Affiliations:** 1 College of Electronic and Optical Engineering, Nanjing University of Posts and Telecommunications, Nanjing 210023, China; 1215022733@njupt.edu.cn (J.L.); 1015020833@njupt.edu.cn (C.D.); Baigang@njupt.edu.cn (G.B.); jiexu@njupt.edu.cn (J.X.); Rqy@njupt.edu.cn (Q.R.); LiJinze@njupt.edu.cn (J.L.); 2 State Key Laboratory of Millimeter Waves, Southeast University, Nanjing 210096, China

**Keywords:** SnO_2_ nanostructure, humidity sensor, nanosphere lithography

## Abstract

Ordered SnO_2_ nanostructures were prepared as humidity sensors by nanosphere lithography with the magnetron sputtering technique. The X-ray diffraction patterns of SnO_2_ nanostructures show that all intense diffraction peaks correspond to the crystallographic planes of SnO_2_. The Atomic Force Microscope (AFM) mage shows that these SnO_2_ nanostructures exhibited a classic honeycomb structure. The resistance of this sensor was measured to show that the resistance of the sensor decreases with an increase from lower relative humidity (RH) to higher RH. Additionally, the longest response/recovery time was 32 s/42 s for 11–96% RH. The hysteresis for the SnO_2_ nanostructure sensor was <5%.

## 1. Introduce

Environmental pollution is currently becoming more and more serious. It does a great harm to human life. Thus, gas sensors have received much attention in recent years [1,2,3,4,5,6,7,8,9]. Humidity sensors are one of the most important sensors, and have been widely used in our day-to-day life. In contrast to other gas sensors, which are used to detect organic vapour gas and hazardous gas, a humidity sensor can provide useful information for health care, living comforts, cultural heritage protection, climate control in green houses, etc. All kinds of nanostructure materials have been extensively studied. Humidity sensors produced from metal oxide semiconductor materials, such as SnO_2_ [10,11], WO_3_ [12,13], and ZnO [14,15], have been applied in a variety of different areas. 

SnO_2_, a wide band gap (~3.7 eV) semiconductor that has unique electrical properties and high physical/chemical stability, is an important material in the field of humidity sensors [16,17]. Tomer et al. found that the SnO_2_/SBA-15 nanocomposite prepared by in-situ method exhibits excellent sensitivity towards change in %RH, as well as good repeatability, short response/recovery time, negligible hysteresis, and great stability [18]. Georgieva and co-workers prepared nanosized thin SnO_2_ layers doped with Te and TeO_2_. This showed perfect humidity sensor characteristics, with very high sensitivity at room temperature, fast response and short recovery period, and good selectivity [19]. Feng et al. reported that SnO_2_ nanostructures with different morphologies had been prepared by a one-step hydrothermal method. The nanosensor, based on 3D hierarchical SnO_2_ dodecahedral nanocrystals, exhibited superior humidity-sensing properties [20]. These SnO_2_ nanostructures are able to provide a regular porosity and a large specific surface area, permitting high accessibility for the water molecules and providing more active sites for the surface physical/chemical interaction between water molecules and the SnO_2_ materials.

Here, we will introduce a successful attempt to synthesize ordered SnO_2_ nanostructures by nanosphere lithography. The nanosphere lithography technique is an inexpensive and efficient method that has been reported for the fabrication of periodic nanostructures [21,22,23]. These nanostructures have a classic honeycomb structure, which can provide a much larger surface area, compared to bulk materials. In our previous work, it was reported that an ordered silicon nanopillar array prepared by nanoshpere lithography exhibited excellent humidity-sensing properties [24,25,26]. Here, the ordered SnO_2_ nanostructure was studied as a new nanostructure material for gas sensors. Room temperature current sensitivity of the ordered SnO_2_ nanostructures sensor was investigated at different values of relative humidity. The result shows that the SnO_2_ nanostructure sensor has good sensing properties with high sensitivity, fast response and short recovery period, and low hysteresis.

## 2. Experiment

**Figure 1:**
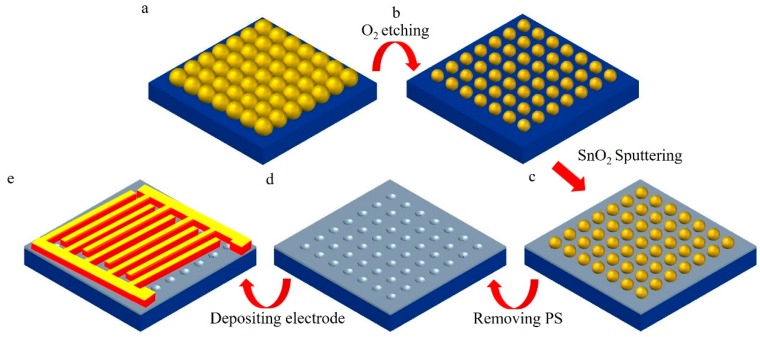
The schematics of the procedure for fabricating the SnO_2_ nanostructure. (**a**) A monolayer of polystyrene nanosphere was fabricated on Si substrate by self-assembly. (**b**) The nanospheres were etching by O_2_. (**c**)The SnO_2_ was sputtering. (**d**) The nanospheres were removed. (**e**) The fork-shaped electrodes were prepared by electron-beam evaporation.

Our approach to production is shown in Figure 1. At first, a monolayer of polystyrene (PS) nanosphere with 220 nm in diameter was fabricated on Si substrate by self-assembly (Figure 1a). The self-assembly method was described in detail in our previous work [25]. Next, the prepared substrate with PS spheres was etched by reaction ion etching (RIE) with oxygen under these conditions: chamber pressure 1 Torr, RF power 20 W, O_2_ flow 20 sccm, and etching time 30 s. O_2_ plasma was applied to shrink the PS nanospheres (Figure 1b). Then, the SnO_2_ film was coated on the PS colloid sphere array by using the magnetron sputtering technique (Figure 1c). The base pressure of the vacuum chamber was 2.0 × 10^−4^ Pa. The SnO_2_ target, with 99.99% purity, was purchased from Beijing Goodwill Metallic Technology Co. The sputtering machine was set to a power of 90 mW, a current of 140 mA, and a 2 min deposition time. During the film deposition, the distance between the PS colloid sphere array substrate and the SnO_2_ target was 20 cm. After sputtering, the sample was put into a solvent, such as methanol or tetrahydrofuran (THF), to remove the PS nanospheres. Then, an ordered SnO_2_ nanostructure was obtained. Subsequently, the performance of thermal annealing for SnO_2_ nanostructure was taken at 1000 °C with oxygen for 1 h. Finally, the fork-shaped electrodes were prepared by electron-beam evaporation (EBV) on the SnO_2_ nanostructure surface. The electrode width and separation distance was 2 mm (Figure 1e). 

The humidity environments were provided by using self-made air-tight glass chambers containing a series of standard aqueous salt solutions (LiCl, Mg(NO_3_)_2_, MgCl_2_, NaCl, KCl and KNO_3_), with the RH being about 11%, 33%, 54%, 75%, 84%, and 96%, respectively. The electrical resistance was monitored by using an Agilent (B1505A) electrometer. All the measurements were carried out under atmospheric pressure and at 25 °C.

## 3. Results and Discussions

**Figure 2:**
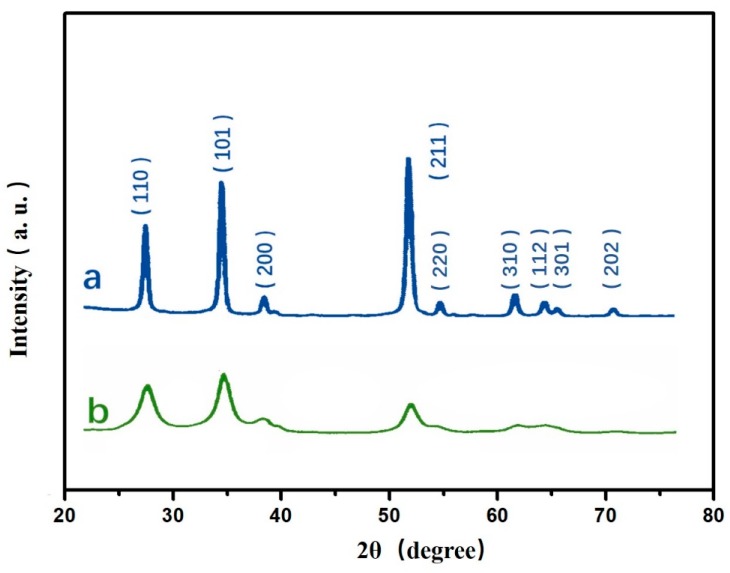
The XRD patterns of SnO_2_ nanostructure film. (a) The SnO_2_ after thermal annealing; (b) as-deposited.

Figure 2 shows the X-ray diffraction patterns of ordered SnO_2_ nanostructure film. Curve a represents the sample with thermal annealing, and curve b is for the sample without thermal annealing. As seen in this figure, nine well-resolved peaks were obtained at 26.8°, 34.4°, 38.1°, 52.4°, 54.9°, 62.3°, 64.6°, 66.1° and 71.8°, corresponding to (110), (101), (200), (211), (220), (310), (112), (301) and (202) planes of SnO_2_, respectively. Furthermore, the full width at half maximum (FWHM) of peak is reduced after thermal annealing. According to the Scherrer equation:

D=kλ/βcosθ,

the size of SnO_2_ nanoparticles is increased after thermal annealing. Here, D is the size of nanoparticles, k is the Scherrer constant, λ is the X-ray wavelength, β is the FWHM and θ is the angle of diffraction. 

**Figure 3:**
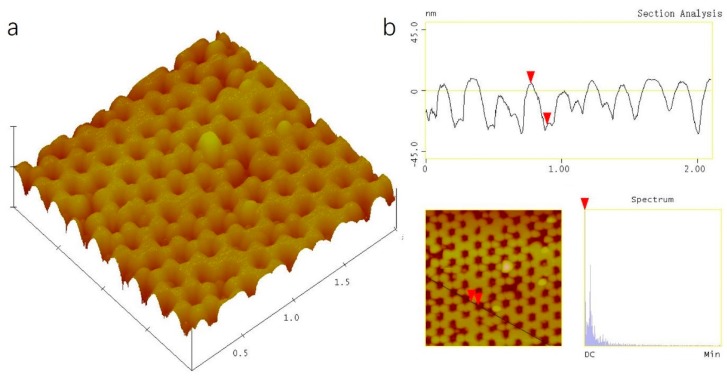
(**a**)The AFM image of SnO_2_ nanostructure. (**b**)The section analysis of SnO_2_ nanostructure.

Figure 3a is the oblique view of AFM image of the SnO_2_ nanostructure. It is shown that the classic honeycomb ordered SnO_2_ nanostructure has been obtained. The period was 220 ± 10 nm, the same as the PS nanosphere size. From Figure 3b, the thickness of the SnO_2_ nanostructure can be seen to be 30 ± 5 nm, and the size of SnO_2_ nanoparticles was 170 ± 10 nm. In the sputtering process, the SnO_2_ film is deposited on the close-packed PS nanosphere array along the perpendicular direction, forming a nanocap array. Meanwhile, the SnO_2_ nanoparticles are able to penetrate the interstice among the three nearest-neighboring PS nanospheres to reach the silicon wafer substrate, creating triangular-shaped SnO_2_ nanoparticle arrays. After removing PS nanospheres, only ordered triangular-shaped SnO_2_ nanoparticles are left on the silicon substrate.

**Figure 4:**
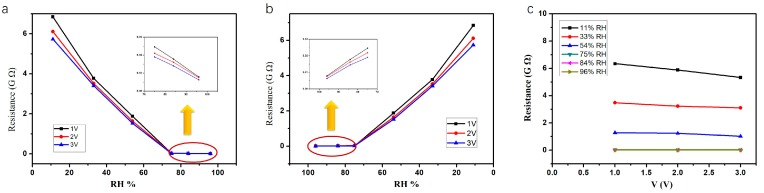
Plot of resistance response of SnO_2_ nanostructures with humidity from 11% to 96% (**a**), and from 96% to 11% (**b**). (**c**) The R-V curves of the SnO_2_ nanostructure with different RHs.

Figure 4a,b shows a resistive-type humidity sensor based on ordered SnO_2_ nanostructures with applied voltage from 1 V to 3 V. It clearly reveals that the resistance of the sensor decreases with an increase from lower RH to higher RH. Figure 4c shows the R-V curves of the SnO_2_ nanostructure at different RHs. It shows a good linear behaviour, which proves that Ohmic laws apply well in this case. By calculation, it was determined that the resistance of this sensor is about 6.54 × 10^9^ Ω at 11% RH, which is 18 times that of 0.36 × 10^9^ Ω at 75% RH and 595 times that of 0.011 × 10^9^ Ω at 96% RH. This proves that water vapour has a strong influence on the conductivity of ordered SnO_2_ nanostructure-based humidity sensors. As observed, the resistance exhibits a large change at RH 75%, which means that more water vapour has been absorbed. In this sensor, the adsorption of water vapour on the surface of SnO_2_ nanostructure sensor is mainly due to the large surface area of the ordered classic honeycomb nanostructure, which is shown in Figure 3a. It is well known that the chemically absorbed oxygen ion (O_2_^−^) is stable on the SnO_2_ surface at room temperature, due to the adsorption energy [27]. The adsorbed oxygen ion on the surface of SnO_2_ nanostructure has been replaced by competitive water molecule absorption. At higher RH, water molecules start to condense, which contributes to the limited surface diffusion of the water molecules. As a result, the depletion layer becomes more and more thin, and the conductivity of the SnO_2_ nanostructure surface increases [28]. 

**Figure 5:**
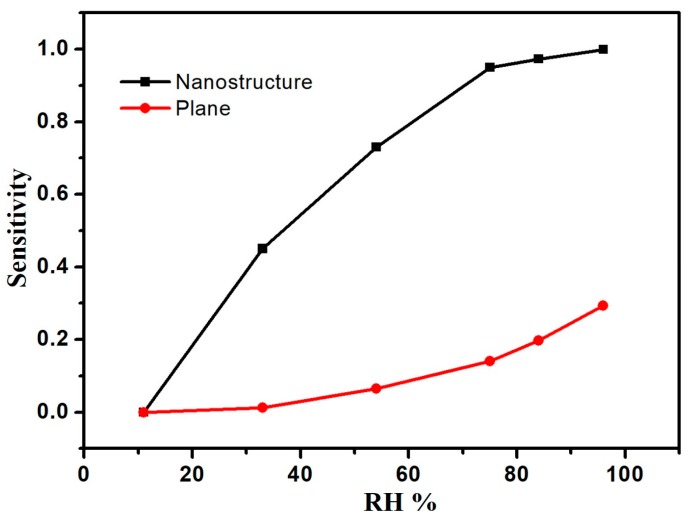
The relation between sensitivity and relative humidity with nanostructure and plane substrate.

**Figure 6:**
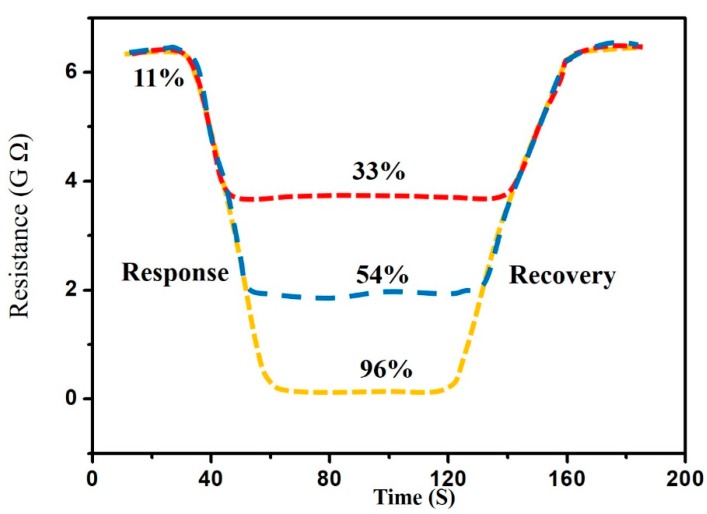
Response/recovery time of the SnO_2_ nanostructure sensor from 11% RH to 96% RH.

K(RH) = (R_1_ − R_2_)/R_1_ is defined as the sensitivity when the applied voltage is 1 V, where R_2_ is the electric resistance at the current humidity and R_1_ is the electric resistance at 11% RH. We also examined the plane substrate as a comparison. Figure 5 shows the relation between sensitivity and relative humidity for the nanostructure and the plane substrate. Obviously, the sensitivity of the nanostructure substrate is better than that of the plane substrate. As seen in Figure 5, the SnO_2_ nanostructure sensor has good sensing properties and shows good linearity with relative humidity at humidity variations between 11% RH and 75% RH. The response and recovery time under different humidity levels was studied, and the results are shown in Figure 6. By measuring, it was found that the average response/recovery times are 18 s/25 s for 11–33% RH, 25 s/33 s for 11–54% RH, 32 s/42 s for 11–96% RH. Table 1 shows the detailed results for the response and recovery time. It was found that the response/recovery time increased with increasing RH, and the remains approximately constant above 75% RH. The average values are 31 s for response time and 40 s for recovery time. It is due to that the surface area was increased by about 50% for this quasi-identical and ordered nanostructure. On the other hand, this provided an effective channel for the absorption and desorption of water molecules. As a result, fast response and recovery times were obtained.

**Figure 7:**
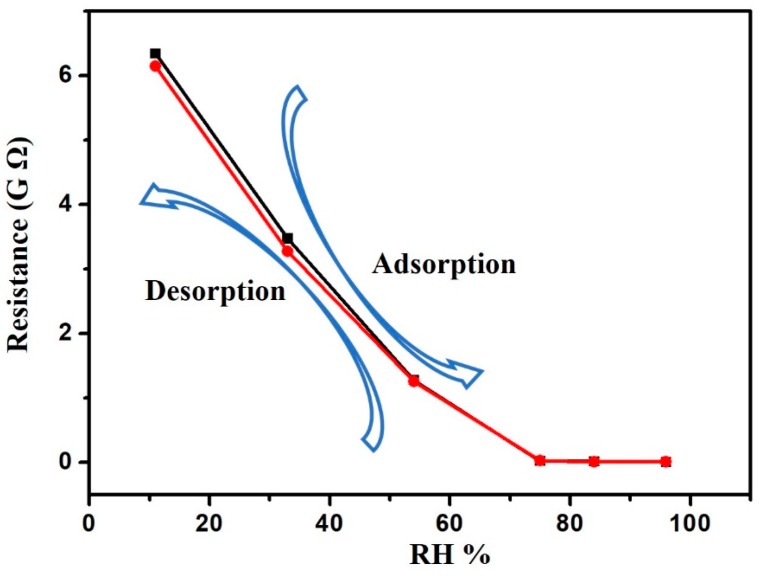
The hysteresis curve showing adsorption/desorption responses.

The hysteresis is an important characteristic of the humidity sensor. Figure 7 shows the hysteresis curve in the process of adsorption and desorption. The lower curve represents the increasing RH (adsorption) process, and the upper curve represents the decreasing RH (desorption) process. As seen in Figure 7, the two curves are very close. The maximum humidity hysteresis is less than 5% at 96% RH, which indicates that the hysteresis for the SnO_2_ nanostructure sensor is small.

## 4. Conclusions

In conclusion, ordered SnO_2_ nanostructures as humidity sensors were fabricated by nanosphere lithography with the magnetron sputtering technique. The resistance of this sensor was measured to show the humidity sensitivity. The results demonstrate that the resistance of the sensor decreases with an increase from lower RH to higher RH. The SnO_2_ nanostructure sensor has good sensing properties and shows good linearity with relative humidity. The longest response/recovery time is 32 s/42 s at humidity variation between 11% RH and 96% RH. The hysteresis of the SnO_2_ nanostructure sensor is less than 5%. These results are due to the large surface area of classic honeycomb ordered nanostructures.

## Figures and Tables

**Table 1:** The response/recovery time of the SnO_2_ nanostructure sensor.

RH	11–33%	11–54%	11–75%	11–84%	11–96%
Response time (s)	18	25	30	31	32
Recovery time (s)	25	33	39	40	42
